# Privacy-Preserving Smart Road-Pricing System with Trustworthiness Evaluation in VANETs †

**DOI:** 10.3390/s21113658

**Published:** 2021-05-24

**Authors:** Qingfeng Zhu, Sai Ji, Jian Shen, Yongjun Ren

**Affiliations:** 1 School of Computer and Software, Nanjing University of Information Science and Technology, Nanjing 210044, China or q_f_zhu@126.com (Q.Z.); 002315@nuist.edu.cn (Y.R.); 2 Suqian University, Suqian 223800, China; jisai@nuist.edu.cn; 3 The Cyberspace Security Research Center, Peng Cheng Laboratory, Shenzhen 518000, China

**Keywords:** intelligent transportation system, road-tolling system, privacy preservation, tolling violations, trustworthiness evaluation

## Abstract

With the advanced development of the intelligent transportation system, vehicular ad hoc networks have been observed as an excellent technology for the development of intelligent traffic management in smart cities. Recently, researchers and industries have paid great attention to the smart road-tolling system. However, it is still a challenging task to ensure geographical location privacy of vehicles and prevent improper behavior of drivers at the same time. In this paper, a reliable road-tolling system with trustworthiness evaluation is proposed, which guarantees that vehicle location privacy is secure and prevents malicious vehicles from tolling violations at the same time. Vehicle route privacy information is encrypted and uploaded to nearby roadside units, which then forward it to the traffic control center for tolling. The traffic control center can compare data collected by roadside units and video surveillance cameras to analyze whether malicious vehicles have behaved incorrectly. Moreover, a trustworthiness evaluation is applied to comprehensively evaluate the multiple attributes of the vehicle to prevent improper behavior. Finally, security analysis and experimental simulation results show that the proposed scheme has better robustness compared with existing approaches.

## 1. Introduction

Vehicular ad hoc networks (VANETs) have attracted keen interest from researchers and industries [1,2,3]. VANETs have been studied in depth over recent years, which has contributed to the construction of smart traffic networks in smart cities. As a promising technology in Intelligent Transportation Systems, VANETs play a key role in avoiding traffic congestion, reducing accidents, decreasing fuel consumption, road safety, and driving comfort [4,5,6]. Optimal road-pricing algorithms force drivers to choose the best routes with less payment, which solves problems in modern society such as exhaust gas pollution.

Since many efforts have focused on developing new tolling methods to better meet the requirements of VANETs in smart cities, road-pricing has evolved into smarter ways, such as the smart road-pricing (SRP) system. Instead of depending on physical equipment, SRP can combine the Global Navigation Satellite System with electronic equipment in vehicles, which makes room for other vehicles and reduces road upkeep [3]. However, properties of decentralization, heterogeneity, and non-trustworthiness in VANETs pose significant challenges in securing message transmission. Therefore, security issues are a priority when deploying this kind of tolling approach, in that malicious vehicles may try to pay less or evade payments. Moreover, the adversary may map the real location of vehicles with user identity to obtain privacy. Therefore, the location of vehicle disclosure may impose heavy threats to drivers.

The trustworthiness of a vehicle is another reference for guaranteeing reliable communication among vehicles or other infrastructure. In [7], a comprehensive evaluation system for vehicles is proposed for the evaluation of various attributes of vehicles. Such an evaluation scheme can provide a real-time update of vehicle status. Various types of communication in VANETs are referenced in [8,9,10]. Vehicles in smart cities can communicate with other vehicles, which is referred to as vehicle-to-vehicle (V2V) communication. Vehicles can also communicate with other infrastructure such as roadside units (RSUs) or the traffic control center (TCC), which is known as vehicle-to-infrastructure (V2I) communication [7]. Additionally, communication between infrastructure can be normal. The communication types in VANETs have been investigated and studied in depth. In our scheme, V2I communication provides a secure channel for vehicles to transmit their geolocation messages to nearby RSUs. At this point, the trustworthiness of vehicles transmitting messages needs to be assessed. Basically, the trustworthiness of a vehicle mainly comes from how many times it has violated tolling rules when using the toll road.

In this paper, an efficient and secure road-pricing system is proposed to better meet the requirements of smart cities. Our purpose is to protect vehicle location privacy during the process of driving while guaranteeing that no driver can perform a tolling violation. In our scheme, only the TCC can trace the real routes of vehicles to protect user privacy disclosure. Moreover, the trustworthiness evaluation of vehicles is applied. The higher the trusted value of a vehicle, the more convenient the services it can obtain, such as priority parking or cheap deals on fuel.

### 1.1. Motivation

The smart road-tolling system has drawn significant attention from researchers and industry, as it succeeds in relieving traffic pressure, reducing fuel consumption, and promoting the construction of eco-friendly cities. Though many smart road-pricing schemes have been proposed, they may not apply practically due to the large communication overhead and redundant operations. To cope with these issues, a privacy-preserving smart road-pricing scheme with trustworthiness evaluation is proposed. First, the term privacy means that a vehicle route needs to be kept secret during communication with RSUs or other vehicles, otherwise malicious entities may track it. Secondly, the identity of the vehicle itself should be protected.

Our contributions: In this paper, an efficient and secure road-pricing system with trustworthiness evaluation is proposed. The contributions of our proposed scheme are as follows:

- A novel effective road-tolling violation scheme is proposed. Smart road-tolling in smart cities can be a challenging task given that tolling violation happens frequently. In this paper, a novel road-tolling violation scheme is proposed. The proposal combines video surveillance cameras (VSCs) and RSUs to detect malicious behavior for a vehicle even if a driver turns off his/her on-board unit (OBU) completely from its vehicle. To be certain, the TCC compares the data collected by VSCs, which are fixed on the pivotal toll road, with the routes collected by RSUs to check whether they are the same.
- A scalable trustworthiness evaluation of the vehicle scheme is investigated. The trustworthiness of a vehicle shows the act of passing through toll roads in the past. In this paper, we present a novel scalable trustworthiness evaluation of vehicle scheme to handle the behaviors of sending false geolocation messages or malicious vehicle users, such as the impersonation of another legitimate vehicle. Therefore, to behave truthfully is the best choice for drivers when using toll roads. The higher the trusted value of a vehicle, the better the access to infrastructure services such as priority parking.
- The detection rate of toll evasion with high efficiency is achieved. On the one hand, though many theoretic smart road-tolling schemes have been proposed, they suffer from a lot of computational overheads. On the other hand, these proposed schemes cannot record tolling violations effectively. That is to say, the schemes which have already been proposed are inefficient. In our scheme, PUF-based VSCs are fixed to pivotal places that can monitor the of passing vehicles accurately. Therefore, the detection rate of toll evasion in our proposed scheme is higher compared to others.

### 1.2. Organization

The structure of this paper is as follows: Section 2 presents related works. Section 3 describes the preliminaries that will be used later. Section 4 provides the system model of our proposed scheme and the design objections. Section 5 introduces a detailed description of our scheme, followed by security analysis in Section 6. Section 7 presents the performance of our scheme and a conclusion is provided after that.

## 2. Related Works

Privacy issues, especially vehicle geolocation data disclosure in road-pricing systems, have drawn widespread attention from researchers over recent years. Numerous solutions have been proposed to achieve security requirements in smart road-pricing.

Vehicle geolocation data can be collected by the OBU and the TCC easily. The encrypted geolocation data is then stored at the TCC, which can reduce the burden of OBU tremendously. Moreover, the TCC can respond in a timely manner when something urgent happens using to the data stored in it [3]. However, such a solution raises another threat after payment information finishes. Chen et al. [11] claimed that this information can be cracked by an external malicious attacker. Therefore, post hoc analysis concerning user traceability based on a user toll payment information scheme has been proposed by them. To avoid violating the location privacy of drivers, Popa et al. [12] proposed a scheme that can be applied to various location-based applications. The authors developed a practical protocol to compute the routing function concerning various tolling, the speed of vehicles, and delay estimation without revealing vehicle geolocation. To ensure the users always pay right tolls, homomorphic commitment [13] has been applied. Random spot-checks with cameras hidden on vehicles are employed to prevent dishonest drivers from cheating on their location. However, an anonymous network is needed to communicate their sensitive traveling data, which imposes heavy overheads on the system. More recently, a group signature [14] toll-pricing system has been proposed by Chen et al. [15] to achieve a balance between vehicle anonymity, computation, and communication overheads. In the proposed scheme, a high-efficiency group signature is deployed to sign each vehicle location before sending them to toll servers. Those vehicles are also grouped by a trusted authority according to criteria such as speed, reputation, and similarity, as per [3,16]. Vehicle privacy in this way can be better protected if proper group management is designed.

With the improvement of smart cities, the location privacy of vehicles plays a significant role in deploying smart electronic systems in VANETs. In 2016, a low-emission zone (LEZ) privacy-preserving road-tolling system was proposed in [17]. The authors divided LEZ into multiple zones, charging various prices according to the topology of the city and the level of congestion. However, the authors in [3] pointed out that vehicles need to authenticate themselves when entering or leaving a zone, which imposes heavy computational overheads. Therefore, a distributed, reliable, and secure pricing system was proposed by Siham Bouchelaghem et al. [3] to better meet the requirements of privacy preservation in SRP. The authors apply a threshold-based control system to discover malicious vehicles who try to cheat on their tolls. Once the accused drivers are tested, a toll server can take relevant measures to punish them. Moreover, the scheme can resist a variety of potential attacks, and the computation and communication overheads are considerably better behaved.

Location-based privacy for vehicles has been investigated actively in recent years. Reza Shokri et al. proposed a *k*-anonymity location privacy preservation scheme [18] in which the real locations of drivers are obfuscated by the construction of cloaking regions. However, Fifi Farouk et al. [6] claimed that this scheme cannot be applied to low-density zones where the users who want to send requests must wait for *k* other users, which may lead to delays and degrade the quality of service. Levente Buttyan et al. [19] proposed that vehicles blind their real identities, which changes with some frequency to solve the problem of privacy disclosure. However, this method may be impractical when applied to long-term communication, because changing frequently may interrupt the quality of correspondence. Recently, Fifi Farouk et al. [6] proposed a location-based service (LBS) to protect the privacy of vehicles using fully homomorphic encryption [20] over advanced encryption standard [21]. However, they do not take road-tolling into consideration.

## 3. Preliminaries

In this section, we present the relative cryptographic primitives used in our scheme.

### 3.1. The Computational Diffie–Hellman (CDH) Assumption

The CDH problem used in our scheme is briefly defined in the following definition. Given an instance (P,aP,bP) where $a,b\in Z_{p}^{*}$, the computational Diffie–Hellman problem (CDH Problem) in a multiplicative group *G* is to compute abP. The success probability of any probabilistic, polynomial-time, 0/1-valued algorithm A to solve CDH problem in *G* can be defined as:

$$\begin{array}{c} Suc_{A,G}^{CDH}=Prob\left[A\left(P,aP,bP\right)=1:a,b\in {}_{R}Z_{q}^{*}\right]. \end{array}$$

The CDH assumption is that for every adversary A in probabilistic polynomial time, the probability of $Suc_{A,G}^{CDH}$ is negligible.

### 3.2. Fuzzy Comprehensive Strategy (FCS)

Driver behavior cannot be determined by a single evaluation accurately, because of the uncertainty and complexity of their actions. Therefore, the fuzzy comprehensive strategy is used to evaluate the trustworthiness of drivers. With such a strategy, multiple attributes and actions are taken into consideration.

#### 3.2.1. Vehicle Behavior Attributes

The behavior attributes of vehicles can be described by a variety of factors, including mileage, timings of vehicle accident records, maximum speed, number of passing tolling spots, and number of toll violations. The trustworthiness of vehicles can be evaluated by the attributes recorded in each vehicle OBU.

#### 3.2.2. Entropy Method

Entropy was originally one of the parameters used to describe the state of matter in thermodynamics. It has been widely used to evaluate multiple-attribute comprehensive evaluation problems since it was introduced into information theory in 1948 by Shannon [22]. According to the degree of variation, information entropy can be used to calculate the entropy weight of attributes. Moreover, entropy weight can also be applied to correct the value to arrive at a more objective weight value.

#### 3.2.3. Comprehensive Attribute Weight

Specifically, comprehensive attribute weight applies various important attributes to vehicle behavior. To obtain objective evaluation results, the attribute weight is not obtained from historical experience, but from the entropy weight mentioned previously. Considering the characteristics of each vehicle’s multiple attributes, it is necessary to illustrate the attribute weight with a comprehensive method $W=\{w_{1},w_{2},\cdots ,w_{n}\}$ where $\sum_{i=1}^{n}w_{i}=1$.

### 3.3. Schnorr Signature

We apply the Schnorr signature [23] to realize our scheme. As with the Elgamal digital signature [24], the Schnorr digital signature is also based on the discrete logarithm problem. The Schnorr scheme minimizes the amount of message computation required to generate the signature. The main work of generating a signature is independent of the message and can be performed when the processor is idle. We chose two large primes p,q, where *q* is the prime factor of p−1 where p,q is assumed to be 1024, and a 160-bit integer respectively. $m\in Z_{p}$ is chosen randomly, and $m^{q}=1\;\mathrm{mod}\;p$. The signature is $\delta_{Schnorr}=\left(r+sY\right)\;\mathrm{mod}\;q$, where $Y=H(M||m^{r})$, *s* is the private key, $r\in Z_{p}$ and satisfies 0<r<q. The public key can be computed by $pk=m^{-s}\;\mathrm{mod}\;p$. To verify the signature, the receiver computes $\xi =m^{\delta_{Schnorr}}pk^{H(M||m^{r})}\;\mathrm{mod}\;p$ and verifies whether $H(M||m^{r})=H(M||\xi )$.

## 4. System and Design Objections

### 4.1. The System Model

**Figure 1:**
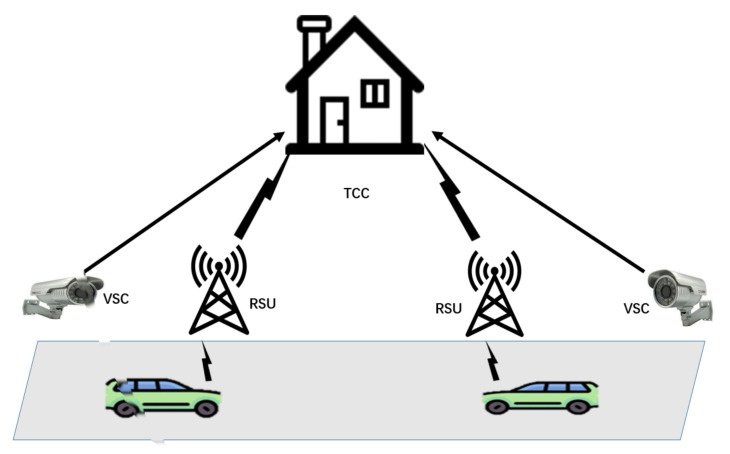
The system model for VANETs.

The system model and design objections of this proposed scheme will be presented in this section. The system model is provided in Figure 1. Please note that for the convenience of display, we only give the model of part of the road for VANETs in Figure 1. In a real scenario, there would be multiple vehicles, RSUs, and VSCs.

- TCC: In our proposed system, the TCC is a fully trusted entity that stores the real identities of vehicles, which are used to track the real driven routes of vehicles if necessary. It also acts as a judge to check whether a vehicle behaves incorrectly by comparing the data collected by VSCs with the data obtained by RSUs in its storage space. The TCC can be managed by a government organization and its computation and communication resources are powerful enough.
- Roadside Unit: As computing and communicating devices, RSUs can receive geolocation information transmitted from vehicles and then transfer them to a cloud server [4]. We assume that the RSUs in our scheme are trusted entities.
- Video Surveillance Camera: As common equipment in modern life, video surveillance cameras (VSCs) play a tremendous role in crime prevention, terrorist detection and obtaining evidence. Equipped with edge computing software units, VSCs have certain computing and storing capabilities. Installed in a pivotal place, the VSC can watch passing vehicles constantly [25]. To ensure security, we adopt the PUF-based VSCs that have been mentioned in [26] for the purpose of resisting various kinds of attacks. Moreover, when regulated by TCC, VSCs behave correctly and are never compromised.
- Vehicle: Equipped with an on-board unit (OBU), vehicles can realize communication and information exchange through a dedicated short-range communication (DSRC) protocol as proposed in [1,2,6,10,27,28]. The vehicle-to-vehicle and vehicle-to-RSU communications are wireless. In our proposed scheme, the vehicles may turn off their OBUs or impersonate a legitimate one to pay less.

### 4.2. The Threat Model

In this part, the threat model of the proposed scheme is presented in detail as follows:

A. An attacker can intercept messages transmitted between VSCs and the TCC, and then may alter, temper, or replay these messages.
B. A malicious vehicle may impersonate another legitimate one to send false geolocation messages for less payment when using toll roads.
C. An adversary may turn off his/her OBU to prevent nearby RSUs from detecting their driving signal to avoid payments.

### 4.3. The Design Goals

Security and privacy issues in VANETs are significant for mutual communication of entities. In this part, detailed design goals are presented as follows:

- Identity privacy preservation: Other malicious vehicles are not able to recover the vehicle’s true identity.
- Message authentication: The TCC can check the validation of messages sent by VSCs, and messages sent by vehicles can also be checked by nearby RSUs.
- Conditional privacy preservation: In the event of a disagreement, the TCC can recover real identities of vehicles by analyzing messages sent by itself. To be specific, a malicious vehicle sends false geolocation message when it uses toll roads to reduce payment.
- Resistance of various kinds of attacks: Our proposed scheme can withstand some frequent attacks such as impersonation attack, modification attack, and man-in-the-middle attack, all of which are harmful to the normal execution of VANETs.

## 5. The Proposed Scheme

In this section, detailed descriptions of our privacy-preserving smart pricing scheme will be presented. The notations used in our scheme is presented in Table 1. Basically speaking, our scheme consists of five stages, named system bootstrapping, VSCs, vehicles and RSUs registration, geolocation message transmission, verification and comparison, trustworthiness evaluation process, and tolling bill.

### 5.1. System Bootstrapping

In this section, the TCC generates the system public parameters. The following operations are carried out by the TCC in our scheme:

- Choosing two large prime numbers p,q and an elliptic curve *E* which is defined by an Equation $y^{2}=x^{3}+ax+b\;\mathrm{mod}\;p$, where a,b∈$F_{p}$.
- A generator *P* is selected in the group *G* which with order *q*. The group *G* consists of all points on the elliptic curve and the infinity point *O*.
- $S\in {Z_{q}}^{*}$ will be selected randomly by the TCC as the system’s private key, and the public key of the system can be therefore computed as $P_{pub}=S\cdot P$.
- Three secure collusion-resistance hash functions are chosen as $H_{1}:G\to Z_{q}$, $H_{2}:{\left\{0,1\right\}}^{*}\to Z_{q}$, $H_{3}:{\left\{0,1\right\}}^{*}\times {\left\{0,1\right\}}^{*}\times G\times {\left\{0,1\right\}}^{*}\to Z_{q}$.
- TCC publicizes the public system parameters $\left\{p,q,a,b,P,P_{pub},H_{1},H_{2},H_{3}\right\}$.

### 5.2. Entity Enrollment

Each PUF-based VSC must preload the system public parameters and register with the TCC, and then the TCC will assign $\left\{I{D^{i}}_{vsc},S\right\}$ to each VSC over a secure channel. Each VSC checks whether the identities are equal to the stored ones. A denial request will be issued if one of them is not equal. Then, each VSC chooses a random number $x_{i}\in {Z_{q}}^{*}$ and computes the pseudonym of VSC which consists of two parts $AID_{vsc_{i}}=\left(AID_{vs{c_{i}}^{1}},AID_{vs{c_{i}}^{2}}\right)$. Later, computing the private key $S{K^{i}}_{vsc}$ of the VSC where $\psi_{i}=H_{2}(AID_{vsc_{i}}\left|\right|T_{i}\left|\right|L_{i})$, $L_{i}$ is the location of the VSC, $T_{i}$ is the timestamp. The specific calculation process is shown in Algorithm 1 lines 1 to 4.

In the process of VSC enrollment, each vehicle is equipped with a tamper-proof device which is preloaded with system public parameters and information $\left\{RI{D^{i}}_{v},S\right\}$ transmitted from the TCC over a secure channel. Then, each vehicle chooses a random number $\hbar_{i}\in {Z_{q}}^{*}$, and computes $\bar{\hbar_{i}}$ and $PI{D^{i}}_{v}$; the private key corresponding to the vehicle anonymity is $S{K^{i}}_{v}$, the public key is $P{K^{i}}_{v}=S{K^{i}}_{v}\cdot P$. At the same time, each vehicle computes ${\Lambda^{i}}_{v}=S{K^{i}}_{v}\cdot P_{pub}$. The specific calculation process is shown in Algorithm 1. The specific calculation process is shown in Algorithm 1 lines 5 to 8.

Each RSU sends the real identity $RID_{RSU_{i}}$ to the TCC over a secure channel, then the TCC chooses a random number $\varepsilon_{i}\in {Z_{q}}^{*}$ and computes $E_{i}$, the private key of corresponding RSU is $S{K^{i}}_{RSU}$, where $T_{i}$ is the timestamp. The public key is $P{K^{i}}_{RSU}$ and compute ${\Lambda^{i}}_{RSU}=S{K^{i}}_{RSU}\cdot P_{pub}$, then the TCC send the $\left\{RID_{RSU_{i}},S{K^{i}}_{RSU},P{K^{i}}_{RSU},E_{i},{\Lambda^{i}}_{RSU}\right\}$ to the corresponding RSU over a secure channel. The specific calculation process is shown in Algorithm 1 lines 9 to 12.
**Algorithm 1** Entities Enrollment**Require:**  $\left\{I{D^{i}}_{vsc},S\right\}$;  1:∀$\lambda \in {Z_{q}}^{*}$, computing $AID_{vsc_{i}{{}^{1}}^{}}=x_{i}\cdot P$, $AID_{vs{c_{i}}^{2}}=RI{D^{i}}_{vsc}⊕H_{1}\left(x_{i}\cdot S\cdot P\right)$;  2:$\psi_{i}=H_{2}(AID_{vsc_{i}}\left|\right|T_{i}\left|\right|L_{i})$;  3:$S{K^{i}}_{vsc}=x_{i}+\psi_{i}\cdot S\;\mathrm{mod}\;q$;  4:**End for**;**Require:**   $\left\{RI{D^{i}}_{v},S\right\}$;  5:∀$\hbar_{i}\in {Z_{q}}^{*}$, computing $\bar{\hbar_{i}}=\hbar_{i}\cdot P$, $PI{D^{i}}_{v}=RID^{i}{}_{v}⊕H_{1}(S\cdot \bar{\hbar_{i}}\left|\right|T_{i})$;  6:Computing vehicle private key $S{K^{i}}_{v}=\hbar_{i}\cdot H_{2}(PI{D^{i}}_{v}\left|\right|\bar{\hbar_{i}}\left|\right|T_{i})+S\left(\;\mathrm{mod}\;q\right)$;  7:Computing vehicle public key $P{K^{i}}_{v}=S{K^{i}}_{v}\cdot P$;  8:**End for**;**Require:**  $RID_{RSU_{i}}$;  9:∀$\varepsilon_{i}\in {Z_{q}}^{*}$, computing $E_{i}=\varepsilon_{i}\cdot P$;  10:Computing RSUs private key $S{K^{i}}_{RSU}=\varepsilon_{i}\cdot H_{2}(RID_{RSU_{i}}\left|\right|E_{i}\left|\right|T_{i})+S\left(\;\mathrm{mod}\;q\right)$;  11:Computing RSUs public key $P{K^{i}}_{RSU}=S{K^{i}}_{RSU}\cdot P$;  12:**End for**;

### 5.3. Geolocation Message Transmission

The geolocation message transmission phase can be divided into two parts, which are separately named VSC evidence generation and transmission, and vehicle route information dissemination, respectively.

In the process of VSC evidence generation and transmission, each VSC which is deployed in a fixed position takes pictures of passing vehicles to record their route information. This information is then transmitted to the TCC. The TCC stores the evidence information transmitted from the VSCs in the database, which is specially designed for storing evidence. The detailed operations are as follows: assuming that a vehicle enters a pricing road, the corresponding road VSC chooses a random number $\omega_{i}\in {Z_{p}}^{*}$, and computes $W_{i}=\omega_{i}\cdot P$, $\alpha_{i}=H_{3}(AID_{vsc_{i}}\left|\right|T_{i}\left|\right|L_{i}\left|\right|W_{i}||LP_{i})$, and generates the evidence signature $\sigma_{i}=S{K^{i}}_{vsc}+\alpha_{i}\cdot \omega_{i}\;\mathrm{mod}\;q$, where $AID_{vsc_{i}}$ is the *i*th anonymity identity of the VSC, $L_{i}$ is the *i*th location information of the vehicle, $LP_{i}$ is *i*th vehicle license plate, which is bound to the driver’s real identity. Then, the VSC sends $\left\{\sigma_{i},AID_{vsc_{i}},T_{i},L_{i},W_{i},LP_{i}\right\}$ to the TCC.

In the process of vehicle route information dissemination, each vehicle sends its geolocation information when entering a toll road. The detailed executions are as follows: for the purpose of privacy protection, each vehicle entering a toll road computes a communication session key with the nearby RSU based on its own private key and the identity of a nearby RSU, where the communication session key can be calculated as $CS{K_{{}_{v_{i}}}}^{RSU_{i}}=S{K^{i}}_{v}\cdot H_{2}(RID_{TCC}||RID_{RSU_{i}}\left|\right|E_{i}\left||S|\right|T_{i})\cdot E_{i}+{\Lambda^{i}}_{v}$, where $RID_{RSU_{i}}$ is the *i*th real identity of RSU. Then, choose a number $z_{i}\in {Z_{q}}^{*}$ randomly, and compute $Z_{i}=z_{i}\cdot P$, $Si{g_{v}}^{i}=S{K^{i}}_{v}+z_{i}\cdot H_{2}(RID_{TCC}||RID_{RSU_{i}}||M)\left(\;\mathrm{mod}\;q\right)$, where $Si{g_{v}}^{i}$ means the message signature generated by the passing vehicles and $M=(L_{i}||T_{i}||PI{D^{i}}_{v})$ represents the message transmitted by a vehicle who enters a toll road at the nearby RSU. To ensure vehicle location privacy, symmetric encryption is adopted to blind the message $C\;=\;E_{K}(M||Si{g_{v}}^{i})$. Then, the generated message $\left\{PI{D^{i}}_{v},C,T_{i},Z_{i},\bar{\hbar_{i}}\right\}$ is transmitted to the nearby RSU.

### 5.4. Verification and Comparison

Upon receiving the message, the RSU operates as Algorithm 2 only if $T_{i}$ is in its valid period: computing the communication session key DK to decrypt the ciphertext *C* to obtain $M||Si{g_{v}}^{i}$. To verify the $Si{g_{v}}^{i}$, the RSU validates whether the Equation is true. Based on the received message, RSUs only need to perform $RI{D^{i}}_{v}=PI{D^{i}}_{v}⊕H_{1}(S\cdot \bar{\hbar_{i}}\left|\right|T_{i})$ to obtain the true identities of passing vehicles. When obtaining the true identities of passing vehicles, RSUs forward $\left\{RI{D^{i}}_{v},L_{i},T_{i}\right\}$ to the TCC over a secure channel for further road-tolling. Later, the TCC verifies the signature to judge whether the messages have been altered by a malicious adversary.
**Algorithm 2** Verification**Require:**   $\left\{PI{D^{i}}_{v},C,T_{i},Z_{i},\bar{\hbar_{i}}\right\}$;  1:Computing $DK=S{K^{i}}_{RSU}\cdot H_{2}(PI{D^{i}}_{v}||RID_{TCC}||RID_{RSU_{i}}\left|\right|Z_{i}\left||S|\right|T_{i})\cdot E_{i}+{\Lambda^{i}}_{RSU}$;  2:Decrypting $M||Si{g_{v}}^{i}=D_{DK}\left(C\right)$;  3:Verifying whether $S{K^{i}}_{RSU}\cdot Si{g_{v}}^{i}\cdot P=DK+Z_{i}\cdot S{K^{i}}_{RSU}\cdot H_{2}(RID_{TCC}||RID_{RSU_{i}}\left|\right|T_{i}\left||M\right)$;  4:**End for**;

The TCC executed the following operations:

- Verification of a single message

Checking the freshness of the $T_{i}$, the TCC rejects the message if the $T_{i}$ is not fresh. The TCC checks whether the Equation $\sigma_{i}\cdot P=AID_{vs{c_{i}}^{1}}+\psi_{i}\cdot P_{pub}+\alpha_{i}\cdot W_{i}$ holds.

- Verification of multiple messages

To speed up verification, many related works have been proposed [29,30,31,32,33]. Therefore, to improve verified efficiency, the small exponent test technique [34,35] is applied. Within such technology, a vector consisting of small random integers can be used to quickly detect any modification in the process of batch verification. Upon receiving multiple messages from VSCs, the TCC verifies the correctness of those messages. First, it checks the freshness of $T_{i}$. Messages merely with the valid $T_{i}$ can be accepted. Second, a vector $\Gamma =\{\tau_{1},\tau_{2},\cdots ,\tau_{n}\}$ is chosen randomly, where $\tau_{i}$ is a small random integer. After that, the TCC verifies whether Equation (1) holds.

(1) $$\begin{array}{c} \left(\sum_{i=1}^{n}\tau_{i}\cdot \sigma_{i}\right)\cdot P=\sum_{i=1}^{n}\left(\tau_{i}\cdot AID_{vs{c_{i}}^{1}}\right)+\left(\sum_{i=1}^{n}\left(\tau_{i}\cdot \psi_{i}\right)\right)\\ \cdot P_{pub}\;+\sum_{i=1}^{n}(\tau_{i}\cdot \alpha_{i}\cdot W_{i}) \end{array}$$

The TCC rejects the messages if the above Equation fails to pass verification; otherwise, the TCC accepts them. Then, the TCC stores the messages in a database 1. For these monitoring messages $\left\{\sigma_{1},AID_{vsc_{1}},T_{1},L_{1},W_{1},LP_{1}\right\}$, $\left\{\sigma_{2},AID_{vsc_{2}},T_{2},L_{2},W_{2},LP_{2}\right\}$, ⋯, $\left\{\sigma_{n},AID_{vsc_{n}},T_{n},L_{n},W_{n},LP_{n}\right\}$ from VSCs, the TCC first perform XOR operations $RID_{vsc}=AID_{vs{c_{i}}^{2}}⊕H_{1}\left(x_{i}\cdot S\cdot P\right)$ to obtain the real identities of each VSC. Afterwards, the TCC stores these monitoring messages $\left\{RID_{vsc_{1}},T_{1},L_{1},LP_{1}\right\}$, $\left\{RID_{vsc_{2}},T_{2},L_{2},LP_{2}\right\}$, ⋯, $\left\{RID_{vsc_{n}},T_{n},L_{n},LP_{n}\right\}$ in database 2. Specifically, the license plate (LP) of each vehicle in database 1 corresponds to the real identities in database 2. We assume that the PUF-based VSCs can never be compromised, and the messages recorded by them can be trusted. Therefore, the messages transmitted from these VSCs which are fixed on pivotal toll roads can be seen as a reference. Then, the TCC compares whether $L_{i}$ stored in database 1 and database 2 are equal using an efficient comparison algorithm in the same period $T_{i}$.

### 5.5. Trustworthiness Evaluation and Tolling Bill

#### 5.5.1. Trustworthiness Evaluation

There are various methods to explore and analyze user behaviors, such as [36,37]. To evaluate the attributes of the vehicle more accurately, a fuzzy comprehensive strategy is adopted in our scheme to analyze each vehicle behavior comprehensively, assuming that the $A=\{a_{1},a_{2},\cdots ,a_{n}\}$ are vehicle *n*-th attributes which can be seen as trustworthiness evaluation indexes. $A^{0}=\left\{{a_{1}}^{0},{a_{2}}^{0},\cdots ,{a_{n}}^{0}\right\}$ denotes the initial attribute weight of each vehicle, and $T^{t}=\left\{t_{1},t_{2},\cdots ,t_{l}\right\}$ means the *l* instances in time $t_{i}$. The following matrix $\bar{A}\;$ is adopted to demonstrate the behavioral attribute clearly.

$$\bar{A}\;=\left[\begin{array}{cccc} A_{11} & A_{12} & \cdots & A_{1n}\\ A_{21} & A_{22} & \cdots & A_{2n}\\ \vdots & \vdots & \ddots & \vdots \\ A_{l1} & A_{l2} & \cdots & A_{ln} \end{array}\right]$$

where $A_{ij}$ denotes the attribute $a_{i}$ in the *j*-th instance. The normalized matrix $\;\bar{\Lambda }\;$ can be obtained by processing the fuzzy matrix $\bar{A}\;$ in the following Equation.

$$\Lambda_{ij}=\left\{\begin{array}{c} \frac{A_{ij}-min\left\{A_{1j},\cdots ,A_{lj}\right\}}{max\left\{A_{1j},\cdots ,A_{lj}\right\}-min\left\{A_{1j},\cdots ,A_{lj}\right\}},A_{ij}\in P^{+};\\ \frac{max\{A_{1j},\cdots ,A_{lj}\}}{max\left\{A_{1j},\cdots ,A_{lj}\right\}-min\left\{A_{1j},\cdots ,A_{lj}\right\}},A_{ij}\in P^{-}. \end{array}\right.$$

where the $P^{+}$ and $P^{-}$ represents the positive and negative attributes, respectively.

$$\;\bar{\Lambda }\;=\left[\begin{array}{cccc} \Lambda_{11} & \Lambda_{12} & \cdots & \Lambda_{1n}\\ \Lambda_{21} & \Lambda_{22} & \cdots & \Lambda_{2n}\\ \vdots & \vdots & \ddots & \vdots \\ \Lambda_{l1} & \Lambda_{l2} & \cdots & \Lambda_{ln} \end{array}\right]$$

As previously mentioned, the entropy method used to demonstrate the uncertainty of things has determined multi-attribute comprehensive evaluation problems in a high-efficiency way. Specifically, the higher uncertainty of the attribute means the higher its weight. According to the normalization of the matrix $\;\bar{\Lambda }\;$, the TCC calculates $\overset{-}{\;\;\Gamma_{ij}}=\frac{A_{ij}}{\sum_{i=1}^{l}A_{ij}}$(i=(1,2,⋯,l),j=(1,2,⋯,n)). For each attribute in j=(1,2,⋯,n), entropy weight can be calculated by the TCC as

(2) $$\begin{array}{c} \overset{-}{w_{j}}=\frac{1-\overset{-}{w}}{n-\sum_{j=1}^{n}\overset{-}{w}}\;\;\;j=\left(1,2,\cdots n\right) \end{array}$$

where $\overset{-}{w}=-\frac{1}{\ln(l)}\sum_{i-1}^{l}\overset{-}{\Gamma_{ij}}\ln\left(\overset{-}{\Gamma_{ij}}\right)$. Therefore, the vehicle’s initial trustworthiness value can be represented as the following Equation

(3) $$\begin{array}{c} IT^{V}=\sum_{j=1}^{n}\frac{\overset{-}{w_{j}}}{n}\sum_{i=1}^{l}\Lambda_{ij}. \end{array}$$

Please note that the $V_{rwd}$ and $V_{psh}$ are the reward and punishment thresholds used to encourage honest vehicle behavior. To be specific, once the initial trustworthiness value is more than $V_{rwd}$, more reward *Q* will be awarded. Otherwise, the initial value will be deducted correspondingly.

The new trusted value of a driver will be increased if the comparison results are equal. By contrast, the new trusted value will be decreased. The following Equation can be used to represent the final trustworthiness value for a vehicle

(4) $$\begin{array}{c} NE{W^{V_{i}}}_{TV}=\left\{\begin{array}{c} \max\left\{0,IT^{V_{i}}-Q\right\},\;\;0\leq IT^{V_{i}}\leq V_{rwd}\\ IT^{V_{i}}\;\;\;\;\;\;\;\;\;\;\;\;\;\;\;\;\;,\;\;\;\;V_{rwd}\leq Q\leq V_{psh}\\ \min\left\{1,IT^{V_{i}}+Q\right\},\;\;\;V_{psh}<Q\leq 1 \end{array}\right. \end{array}$$

#### 5.5.2. Toll Bill

The final toll bill will be generated based on the trusted value $NE{W^{V_{i}}}_{TV}$ at the end of pricing period and then be sent to the drivers who use the toll road. For each vehicle, the TCC calculates the fee $g({T_{i}}^{j},{L_{i}}^{j})$ with the billing function, where *j* represents the vehicle using the toll road for *j* times. The final total costs of the *i*-th vehicle can be represented by $Bil{l^{i}}_{v}=\sum_{j=1}^{n}g({T_{i}}^{j},{L_{i}}^{j},NEW_{}{{{}^{V_{i}}}_{TV}}^{j})$, where the vehicle trusted value $NE{W^{V_{i}}}_{TV}$ is incorporated. To guarantee the integrity and authenticity of the bill, the TCC encrypts the bill using the public key of a vehicle, and then signs it using the private key of the TCC. Detailed operations of the TCC are as follows: randomly chosen numbers $\lambda \in {Z_{q}}^{*}$, and $\left(\Phi_{1},\Phi_{2}\right)$ are computed where $\Phi_{1}=\lambda \cdot P,\Phi_{2}=\lambda \cdot P{K^{i}}_{v}$. Afterwards, $ℜ=Bil{l^{i}}_{v}\cdot X_{\Phi_{2}}\;\mathrm{mod}\;p$ and $\eta =\lambda -P_{pub}\cdot ℜ\;\mathrm{mod}\;q$ are calculated where $X_{\Phi_{2}}$ means the horizontal axis of $\Phi_{2}$. Finally, the TCC generates the bill’s signature $\sigma_{Bil{l^{i}}_{v}}=\langle ℜ||\eta ||\Phi_{1}\rangle$. The detailed generation process of the toll bill is provided in Algorithm 3.
**Algorithm 3** Generating Tolling Bill Signature**Require:**  ${{\langle {T_{i}}^{j},{L_{i}}^{j}\rangle }^{n}}_{j=1},P{K^{i}}_{v}$;  1:$Bil{l^{i}}_{v}\leftarrow 0$;  2:**for all**1≤j≤n**do**;  3:$Bil{l^{i}}_{v}\leftarrow Bil{l^{i}}_{v}+g\left({T_{i}}^{j},{L_{i}}^{j},NEW_{}{{{}^{V_{i}}}_{TV}}^{j}\right)$;  4:**End for**;  5:Randomly choose $\lambda \in {Z_{q}}^{*}$;  6:$\Phi_{1}\leftarrow \lambda \cdot P$;  7:$\Phi_{2}\leftarrow \lambda \cdot P{K^{i}}_{v}$;  8:$ℜ=Bil{l^{i}}_{v}\cdot X_{\Phi_{2}}\;\mathrm{mod}\;p$;  9:$\eta =\lambda -P_{pub}\cdot ℜ\;\mathrm{mod}\;q$;  10:$\sigma_{Bil{l^{i}}_{v}}=\langle ℜ||\eta ||\Phi_{1}\rangle$;  11:**return**$\sigma_{Bil{l^{i}}_{v}}$;

Only those bills that pass the signature verification can be seen as a legitimate bill. For this purpose, the vehicle checks whether the Equation $ℜ\cdot P_{pub}+\eta \cdot P=S{K^{i}}_{v}\cdot {\Phi_{1}}^{\prime }$ holds. The vehicle accepts the bill if the verification holds, and then recovers the toll bill $Bil{l^{i}}_{v}$ by computing $Bil{l^{i}}_{v}=ℜ\cdot {X^{-1}}_{{\Phi_{2}}^{\prime }}\;\mathrm{mod}\;p$. Otherwise, the vehicle rejects it. The detailed signature verification process of the toll bill is provided in Algorithm 4.
**Algorithm 4** Verification of the toll bill for each vehicle**Require:** $\sigma_{Bil{l^{i}}_{v}},S{K^{i}}_{v}$;  1:${\Phi_{1}}^{\prime }\leftarrow ℜ\cdot P_{pub}+\eta \cdot P$;  2:${\Phi_{2}}^{\prime }\leftarrow S{K^{i}}_{v}\cdot {\Phi_{1}}^{\prime }$;  3:**if (${\Phi_{2}}^{\prime }={\Phi_{1}}^{\prime }$) then**  4:$Bil{l^{i}}_{v}\leftarrow ℜ\cdot {X^{-1}}_{{\Phi_{2}}^{\prime }}\;\mathrm{mod}\;p$;  5:**return**$Bil{l^{i}}_{v}$;  6:**else**  7:**return** invalid signature;  8:**end if**;

## 6. Security Analysis

In this section, security analysis is presented to show that how our proposed scheme can satisfy our design objections and resist some attacks.

### 6.1. Correctness

- The signature messages generated by VSCs are correct and can resist threat model A mentioned in Section 4.2 if the system security parameters are correctly generated. The proof is as follows:

With system security parameters and signature messages generated by VSCs and vehicles, our proposed scheme can be proved to be correct as per the following Equation.

(5) $$\begin{array}{cc} \left(\sum_{i=1}^{n}\tau_{i}\cdot \sigma_{i}\right)\cdot P & =\left(\sum_{i=1}^{n}\tau_{i}\cdot (S{K^{i}}_{vsc}+\alpha_{i}\cdot \omega_{i})\right)\cdot P\\ \; & =\sum_{i=1}^{n}(\tau_{i}\cdot AID_{vs{c_{i}}^{1}})+\sum_{i=1}^{n}(\tau_{i}\cdot \psi_{i}\cdot P_{pub})\\ & +\sum_{i=1}^{n}(\tau_{i}\cdot \alpha_{i}\cdot W_{i})\\ \; & =\sum_{i=1}^{n}(\tau_{i}\cdot AID_{vs{c_{i}}^{1}})+\sum_{i=1}^{n}(\tau_{i}\cdot \psi_{i})\cdot P_{pub}\\ & +\sum_{i=1}^{n}(\tau_{i}\cdot \alpha_{i}\cdot W_{i}) \end{array}$$

- The signature messages generated by vehicles are correct if the system security parameters are correctly generated.

With system security parameters and signature messages generated by vehicles, our proposed scheme can be proved to be correct as per the following Equation.

(6) $$\begin{array}{cc} \; & S{K^{i}}_{RSU}\cdot Si{g_{v}}^{i}\cdot P\\ \; & =S{K^{i}}_{RSU}\cdot \left(S{K^{i}}_{v}+z_{i}\cdot \Theta_{1}\right)\cdot P\\ \; & =S{K^{i}}_{RSU}\cdot S{K^{i}}_{v}\cdot P+S{K^{i}}_{RSU}\cdot z_{i}\cdot P\cdot \Theta_{1}\\ \; & =S{K^{i}}_{RSU}\cdot \Theta_{2}\cdot P+S{K^{i}}_{RSU}\cdot Z_{i}\;\cdot \Theta_{1}\\ \; & =DK+S{K^{i}}_{RSU}\cdot Z_{i}\cdot \Theta_{1} \end{array}$$

where $\Theta_{1}=H_{2}(RID_{TCC}||RID_{RSU_{i}}\left|\right|T_{i}\left||M\right)$, and $\Theta_{2}=\hbar_{i}\cdot H_{2}(PI{D^{i}}_{v}\left|\right|\bar{\hbar_{i}}\left|\right|T_{i})+S$.

**Theorem** **1.** *The signatures generated by VSCs and vehicles are unforgeable if all system parameters are created correctly.*

**Proof** **of** **Theorem** **1.** Based on the threat model and design objections, the security model of our proposed scheme is defined by a game, which involves the interaction between a challenger ***C*** and an adversary ***A***. Assuming there exists an adversary ***A*** who can forge a valid signature, a challenger ***C***, who is able to solve the hardness of the *DL* problem with a non-negligible advantage, uses ***A*** as a subroutine. The detailed proof of our scheme is similar to the one in [38]. Due to space limitation, we omit it in this paper.    □

### 6.2. Security Discussion

- Identity privacy preservation

The real identities of vehicles $RID^{i}{}_{v}$ are blinded by computing $\bar{\hbar_{i}}=\hbar_{i}\cdot P$ and $PI{D^{i}}_{v}=RID^{i}{}_{v}⊕H_{1}(S\cdot \bar{\hbar_{i}}\left|\right|T_{i})$. To obtain the real identities $RID^{i}{}_{v}$ from $PI{D^{i}}_{v}$, the adversary must compute $S\cdot \bar{\hbar_{i}}=S\cdot \hbar_{i}\cdot P=\hbar_{i}\cdot P_{pub}$ from $P_{pub}=S\cdot P$. Therefore, due to the hardness of the CDH problem defined previously, we show that our proposed scheme can provide identity privacy preservation.

- Message authentication

In our scheme, the signature messages $\left\{\sigma_{i},AID_{vsc_{i}},T_{i},L_{i},W_{i},LP_{i}\right\}$ where $\sigma_{i}=S{K^{i}}_{vsc}+\alpha_{i}\cdot \omega_{i}\;\mathrm{mod}\;q$, $Si{g_{v}}^{i}=S{K^{i}}_{v}+z_{i}\cdot H_{2}(RID_{TCC}||RID_{RSU_{i}}\left|\right|T_{i}||M)\;\mathrm{mod}\;q$ generated by VSCs and vehicles during passing toll road can be checked by the TCC and RSUs, and the correctness of the Equation verification is shown in Section 6.1. Using Section 6.1, the threat model B can be avoided in our proposed scheme.

- Conditional privacy preservation

The real identities of vehicles $RI{D^{i}}_{v}$ are covered up by $PI{D^{i}}_{v}=RID^{i}{}_{v}⊕H_{1}(S\cdot \bar{\hbar_{i}}\left|\right|T_{i})$ where $\bar{\hbar_{i}}=\hbar_{i}\cdot P$. The TCC can perform an XOR operation as $RI{D^{i}}_{v}=PI{D^{i}}_{v}⊕H_{1}(S\cdot \bar{\hbar_{i}}\left|\right|T_{i})$ using the system master private key *S* in an emergency or in the event of a disagreement to obtain vehicle real identities.

- Resisting impersonation attacks

Adversaries who want to impersonate a legitimate actor to reduce payment must generate a message $\left\{PI{D^{i}}_{v},C,T_{i},Z_{i},\bar{\hbar_{i}}\right\}$ satisfying the Equation $S{K^{i}}_{RSU}\cdot Si{g_{v}}^{i}\cdot P=DK+Z_{i}\cdot S{K^{i}}_{RSU}\cdot H_{2}(RID_{TCC}||RID_{RSU_{i}}\left|\right|T_{i}\left||M\right)$. As is shown in Section 6.1, RSUs can identify such an attack easily by checking whether Equation (6) holds. Therefore, any adversaries cannot impersonate a legitimate vehicle.

- Resisting modification attacks

$\left\{\sigma_{i},AID_{vsc_{i}},T_{i},L_{i},W_{i},LP_{i}\right\}$ is a signature generated by VSCs fixed on pivotal toll roads. According to Theorem 1, the TCC can easily identify whether the signature $\left\{\sigma_{i},AID_{vsc_{i}},T_{i},L_{i},W_{i},LP_{i}\right\}$ generated by VSCs has been modified by checking the Equation $\sigma_{i}\cdot P=AID_{vs{c_{i}}^{1}}+\psi_{i}\cdot P_{pub}+\alpha_{i}\cdot W_{i}$.

- Resisting man-in-the-middle attacks

Based on message authentication among entities such as VSCs, vehicles, and the TCC, we know that our proposed scheme can provide authentication for participants. Therefore, a man-in-the-middle attack can by resisted in our proposed scheme.

## 7. Performance Evaluation

In this section, the performance analysis of our proposed scheme is presented. To better demonstrate our proposed protocol intuitively, implementation with a pairing-based cryptography (PBC) library https://crypto.stanford.edu/pbc/ (accessed on 19 May 2021) and GNU Multiple Precision Arithmetic library on a Linux system using an Intel Core i5-9500 at a frequency of 3.0 GHz, and 8 GB of RAM are provided.

**Figure 2:**
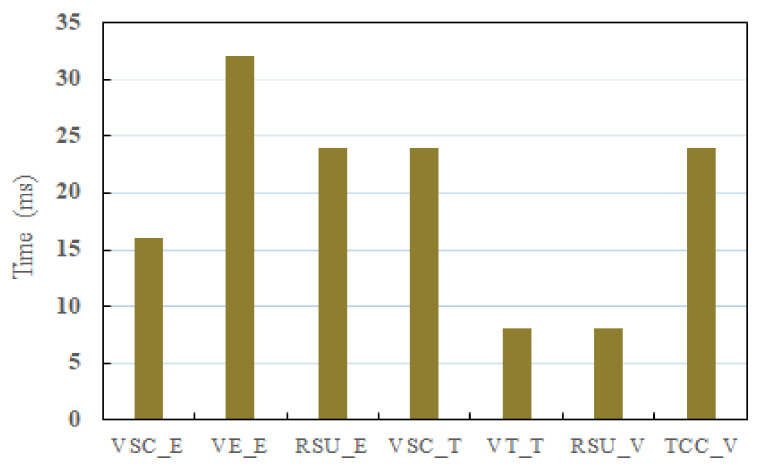
The overhead comparison among different phases of our scheme.

As illustrated in Figure 2, seven phases are separately named VSC enrollment, vehicle enrollment, RSU enrollment, message transmission of VSCs, message transmission of vehicles, message verification of RSUs, and TCC, respectively. It is not difficult to see that the time cost of vehicle enrollment is more than the other entities. Essentially, the vehicle needs to execute a point multiplication related to ECC, two additive operations, and three multiplication operations on $Z_{p}$ in this phase. However, this takes a lot of time, so this phase can be executed offline ahead of time. Therefore, the amount of system time cost on execution is not burdensome.

**Figure 3:**
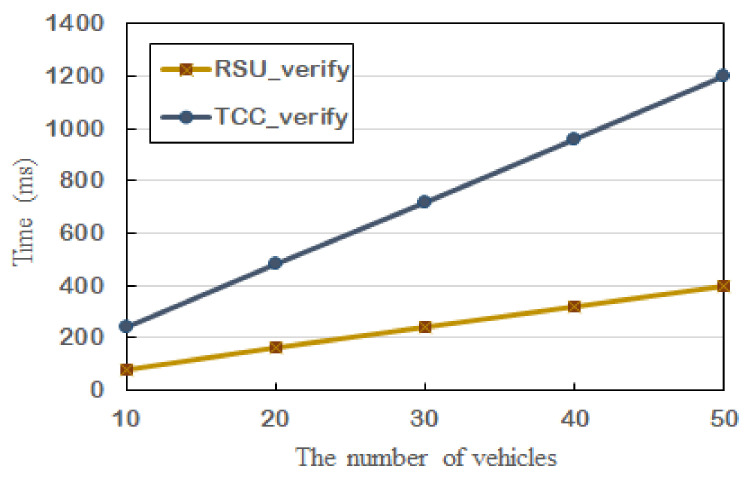
The time cost of signature verification.

Figure 3 presents the time cost of signature verification. With the increase of the number of vehicles, the computation overheads of the RSU and TCC are increased. Essentially, each vehicle sends geolocation messages to the RSU and each VSC sends surveillance messages to the TCC, which leads to increased overheads. The reason that the time cost of the TCC is higher than the RSUs is that the TCC needs to execute three multiplication operations related to the ECC and an additive operation on $Z_{p}$.

**Figure 4:**
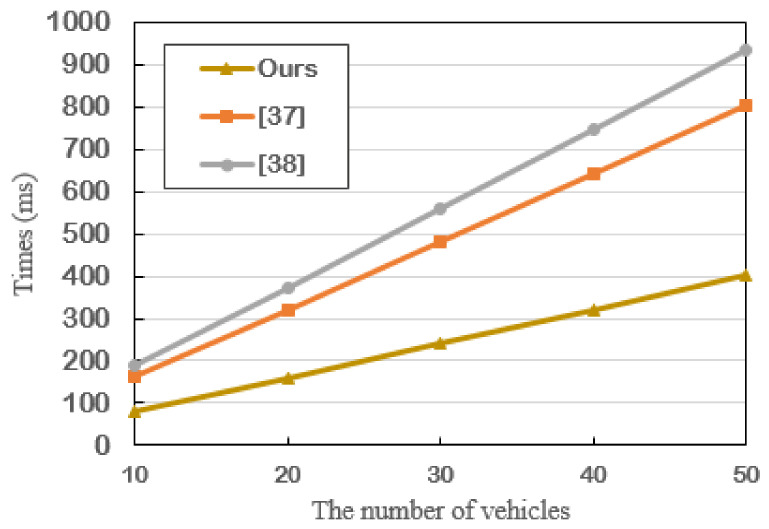
The time cost of signature verification of different schemes.

To illustrate the superiority of our proposed scheme, the time costs of signature verification of different schemes are presented in Figure 4. Clearly, performance analysis shows that our scheme is much better than [38,39], i.e., the computation overheads in our scheme are much lower. Without using bilinear pairing, our proposed scheme can better meet the requirements of resource constraint in VANETs.

**Figure 5:**
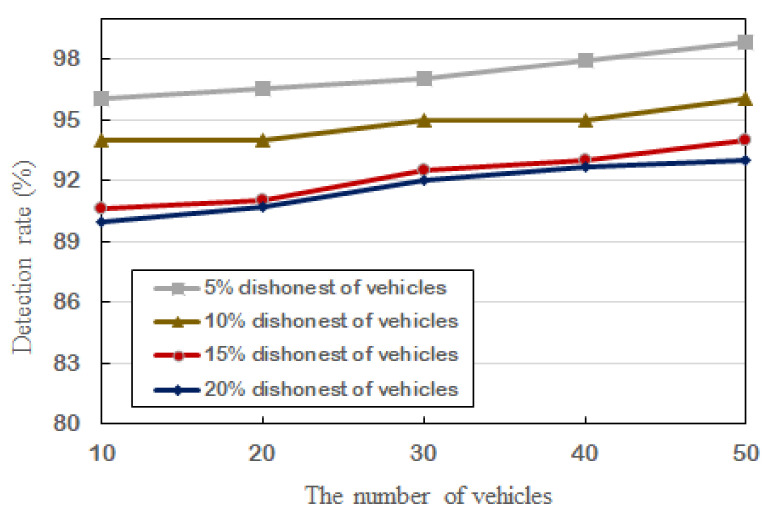
The detection rate and corresponding vehicles.

To cope with drivers who want to reduce payments or pay no bill by turning off his/her OBU, the performance of detection rates is evaluated. As shown in Figure 5, with the increase in vehicles, the detection rate increases slowly. Please note that the lowest detection rate of our scheme is still more than 89%, though the number of vehicles is 50.

In fact, no more than 50 vehicles will pass through the toll road at the same time in the existing highway section. Therefore, our proposed scheme can be easily applied in practical application.

## 8. Conclusions

In this paper, a novel privacy-preserving road-pricing system with a trustworthiness evaluation scheme is proposed. In the scheme, we combine cryptographic primitives and our unique comparison method to force passing vehicles to behave honestly as much as possible. The PUF-based VSCs, which can resist various attacks such as man-in-the-middle, are used to record the real geolocation and corresponding time. Meanwhile, messages generated by the vehicle itself can be received by nearby RSUs and then be forwarded to the TCC, which compares whether they are equal. Moreover, a novel fuzzy comprehensive strategy trustworthiness evaluation approach is designed and applied to our proposed scheme to record vehicle misbehavior. Finally, sufficient theoretical and experimental analysis yields better performance in security and efficiency in comparison with previous schemes.

## Figures and Tables

**Table 1:** Notations in our scheme.

Symbol	Description
*G*	An additive group with order *q*
*E*	An elliptic curve $y^{2}=x^{3}+ax+b\;\mathrm{mod}\;p$
p,q	Represent two large prime numbers
*S*	The master key generated by traffic control center
*P*	The group generator
$P_{pub}$	The system public key, where $P_{pub}=S\cdot P$
$AID_{vsc_{i}}$	The pseudonym of VSC including $\left(AID_{vs{c_{i}}^{1}},AID_{vs{c_{i}}^{2}}\right)$
$S{K^{i}}_{vsc}$	The private key of the VSC
$S{K^{i}}_{v}$	The private key of the vehicle
$RI{D^{i}}_{V}$	The *i*-th true identity of the vehicle
$PI{D^{i}}_{V}$	The *i*-th pseudonym identity of the vehicle
$RI{D^{i}}_{VSC}$	The true identity of a VSC
$PI{D^{i}}_{VSC}$	The *i*-th pseudonym identity of a VSC
$E_{K}\left(\right)/D_{K}\left(\right)$	The symmetric encryption/decryption function
$H_{1}$	Hash function $H_{1}:G\to Z_{q}$
$H_{2}$	Hash function $H_{2}:{\left\{0,1\right\}}^{*}\to Z_{q}$
$H_{3}$	Hash function $H_{3}:{\left\{0,1\right\}}^{*}\times {\left\{0,1\right\}}^{*}\times G\times {\left\{0,1\right\}}^{*}$
	$\to Z_{q}$
*n*	The total number of vehicles passing a toll road
	in a period time
⊕	Represent the exclusive-OR-operation
||	The information concatenation operation
